# Cross-Sectional Analysis of the Microbiota of Human Gut and Its Direct Environment in a Household Cohort with High Background of Antibiotic Use

**DOI:** 10.3390/microorganisms9102115

**Published:** 2021-10-08

**Authors:** Bich Vu Thi Ngoc, Hai Ho Bich, Gianluca Galazzo, Dung Vu Tien Viet, Melissa Oomen, Trang Nghiem Nguyen Minh, Hoang Tran Huy, Hindrik Rogier van Doorn, Heiman F. L. Wertheim, John Penders

**Affiliations:** 1 Welcome Trust Major Asia Programme, Oxford University Clinical Research Unit, Ha Noi 100000, Vietnam; bichvtn@oucru.org (B.V.T.N.); haihb@oucru.org (H.H.B.); dungvtv@oucru.org (D.V.T.V.); trangnnm@oucru.org (T.N.N.M.); rvandoorn@oucru.org (H.R.v.D.); 2 Department of Medical Microbiology, Radboudumc Center for Infectious Diseases, Radboud University Medical Center, 5129 Nijmegen, The Netherlands; Heiman.Wertheim@radboudumc.nl; 3 School for Nutrition and Translational Research in Metabolism (NUTRIM) and Care and Public Health Research Institute (Caphri), Department of Medical Microbiology, Maastricht University Medical Center, 3112 Maastricht, The Netherlands; g.galazzo@maastrichtuniversity.nl (G.G.); melissa.oomen@mumc.nl (M.O.); 4 National Institute of Hygiene and Epidemiology, Ha Noi 100000, Vietnam; thh@nihe.org.vn; 5 Center for Tropical Medicine, Nuffield Department of Clinical Medicine, University of Oxford, Oxford OX3 7BN, UK

**Keywords:** microbiota, One Health, Vietnam, environmental microbiota, metagenomics, antibiotic resistance

## Abstract

Comprehensive insight into the microbiota of the gut of humans and animals, as well as their living environment, in communities with a high background of antibiotic use and antibiotic resistance genes is scarce. Here, we used 16S rRNA gene sequencing to describe the (dis)similarities in the microbiota of feces from humans (*n* = 107), domestic animals (*n* = 36), water (*n* = 89), and processed food (*n* = 74) in a cohort with individual history of antibiotic use in northern Vietnam. A significantly lower microbial diversity was observed among individuals who used antibiotics in the past 4 months (*n* = 44) compared to those who did not (*n* = 63). Fecal microbiota of humans was more diverse than nonhuman samples and shared a small part of its amplicon sequence variants (ASVs) with feces from animals (7.4% (3.2–9.9)), water (2.2% (1.2–2.8)), and food (3.1% (1.5–3.1)). Sharing of ASVs between humans and companion animals was not associated with the household. However, we did observe a correlation between an Enterobacteriaceae ASV and the presence of extended-spectrum beta-lactamase *CTX-M-*group*-*2 encoding genes in feces from humans and animals (*p* = 1.6 × 10^−3^ and *p* = 2.6 × 10^−2^, respectively), hinting toward an exchange of antimicrobial-resistant strains between reservoirs.

## 1. Introduction

Microbiotas are complex and diverse ecological communities of microorganisms that are specific to a particular habitat such as specific human or animal body sites (e.g., the intestinal tract) or natural environments [1,2,3]. The native microbial composition is not only strongly associated with host factors [2] but also with geographic and environmental variables [4,5]. Physical interactions between hosts and their natural environment may also alter the microbiota diversity and composition [6]. Recent work on the microbiota of humans and animals has suggested that, due to interactions, such as contact with natural environments and co-housing conditions, individuals may share specific bacterial strains or have a more similar microbiota with each other and their environments [7]. From a One Health perspective, insights into the interconnections among humans, animals, and their environmental microbiome are important to further our understanding on the dynamics in bacterial communities, including potential pathogenic and antimicrobial strains [8,9]. The consumption of antibiotics has a profound effect on distortion of the microbiota of humans and animals, resulting in loss of diversity and keystone species [10,11,12]. A study conducted among 12 healthy volunteers Caucasian males aged between 18 and 45 years showed that administrating antibiotics, including meropenem, gentamicin, and vancomycin, to healthy individuals led to an enrichment of antibiotic resistance genes (ARGs) and loss of butyrate-producing bacteria [13]. In addition, repeated exposure to antibiotics not only drove the microbiome into a new steady state that was different from the original state but also increased the survival and colonization potential of antibiotic-resistant bacteria [14,15]. Furthermore, antibiotic pollution into natural environments also influences soil and aquatic microbial ecosystems [16].

To date, most research on the effects of antibiotics has, however, been focused on microbial perturbations among study subjects without correlation to other co-habitats or vice versa [17]. To better understand host–microbiota relationships, it is important to place the research of the microbiota in the broader environmental–ecological context [18]. Furthermore, it is of interest how the microbiota adapts in settings where antibiotic exposure is expectedly frequent. Few studies have made such an attempt to characterize and compare the gut microbiota among individuals and their environment in a community setting with high antibiotic use [17,19].

As an agricultural country, with nearly 50% of the population consisting of farmers in rural areas according to the report of general statistics office in 2019, Vietnam has a history of using antibiotics in treatment and prevention in humans, animals, and aquaculture [20,21,22]. The impact of antibiotic use in Vietnam on both community and hospital settings has been evaluated in many different aspects, including economic impacts, water pollution, and the diversity and abundance of the resistome [23,24,25]. However, there are still large gaps in our understanding of the human microbiota and its interactions with nonhuman microbial ecosystems. Here, we used 16S ribosomal RNA gene amplicon sequencing to characterize the microbiota of a cross-sectional collection of stool samples from humans and their domestic animals, as well as their food and water, taken from a rural community cohort in Ha Nam, Vietnam. We describe the microbial diversity, composition, and community structure of feces from humans and domestic animals, as well as their environment, in association with antibiotic consumption and a high background of antibiotic resistance genes.

## 2. Material and Methods

### 2.1. Community Setting, Study Design, and Sample Collection

The demographics of this complete cohort were described in our previous study, which was designed to explore the circulation of ARGs in humans, animals, and their environment [26] (see Supplementary Table S1).

For the present study, a subset of 44/80 participating households was selected: 23 households in which at least one member used any antibiotic in the 4 months prior to sample collection and 21 households in which no antibiotic use was reported prior to sampling. We used feces from humans (*n* = 107) and domestic animals (*n* = 36), processed foods (*n* = 74), and water samples (water used for cooking and/or washing and irrigation) (*n* = 89) collected at a single timepoint (at the fourth month of study—M4) collected from these selected households (HHs) to analyze the microbiota using 16S rRNA gene amplicon sequencing.

In this study, samples were collected from 44 households (HHs) residing in seven villages within 8.1 square kilometers. Lifestyles in these villages are very similar with diets dominated by rice, vegetables, and meat. The frequency of meat consumption was dependent on the economic condition of HHs. To evaluate the geographical impact on the microbiota, particularly in order to identify whether individuals living closer together shared more ASVs or not, we separated the 44 HHs into clusters based on their locations. We used partitioning clustering with the k-medoids algorithm, which enabled us to find out two clusters in 44 HHs based on the distance from HH locations (detected by their latitude and longitude) to the center of each cluster. The numbers of participating individuals living in cluster 1 with 23 HHs and cluster 2 with 21 HHs were 49 and 58, respectively (see Supplementary Figure S1 and Supplementary Table S2).

### 2.2. Methods

#### 2.2.1. DNA Extraction and 16S rRNA Amplicon Sequencing

Metagenomic DNA was isolated as described previously [26]. In brief, repeated bead beating (RBB) combined with column-based purification was used to extract DNA from human and animal fecal samples according to protocol Q of the International Human Microbiome Standards consortium. For isolation of microbial DNA from water samples, 100 mL of collected water was filtered through 0.22 μm mixed cellulose ester membrane filters (Sartorius, Göttingen, Germany) to capture bacteria. One-quarter of the filters were used for metagenomic DNA extraction using the QIAGEN DNeasy Power Water kit according to the manufacturer’s protocol (Qiagen, Hilden, Germany). Upon isolation, DNA concentrations were determined using the Quan-iT PicoGreen dsDNA assay (Invitrogen, Carlsbad, CA, USA).

Amplicon libraries and sequencing were performed as described previously [27]. Briefly, the V4 region of the 16S rRNA gene was PCR-amplified from each DNA sample in triplicate using the 515 f/806r primer pair [28]. Pooled amplicons from the triplicate reactions were purified using AMPure XP bead purification (Beckman Coulter, Brea, CA, USA) according to the manufacturer’s instructions, eluted in 25 µL of 1× low TE (10 mM Tris-HCl, 0.1 mM EDTA, pH 8.0), and subsequently quantified by Quant-iT PicoGreen dsDNA reagent kit (Invitrogen, New York, NY, USA) using a Victor3 Multilabel Counter (Perkin Elmer, Waltham, MA, USA). Amplicons were mixed in equimolar concentrations to ensure equal representation of each sample and were sequenced on an Illumina MiSeq instrument using the V3 reagent kit (2 × 250 cycles) (Illumina, San Diego, CA, USA).

#### 2.2.2. Bioinformatics Analysis

Raw reads from the 16S rRNA amplicon sequencing were demultiplexed and quality-controlled using analysis package QIIME2-2019.7 (Quantitative Insights Into Microbial Ecology 2-2019.7) [29]. We used the DADA2 pipeline for sequence quality control and feature table construction [30] (via q2-dada2). We truncated sequences at position 200 of forward reads and at position 140 of reverse reads to remove low-quality regions of reads while maintaining sufficient overlap between forward and reverse reads. The high-quality reads resulting from denoising and chimera-filtering steps were clustered into a table of amplicon sequence variants (ASVs) [31]. An ASV refers to a single DNA sequence recovered from a high-throughput marker gene analysis upon removal of erroneous sequences generated during PCR and sequencing and is, therefore, a higher-resolution analog of the traditional operational taxonomic units [30]. Samples with a sequencing depth below 5000 reads were excluded prior to the downstream analyses, and all ASVs with a relative abundance less than <0.0001% were discarded. We further removed contaminant ASVs annotated as mitochondria and chloroplast, and we generated a tree for phylogenetic diversity analyses using FastTree2 [32]. Taxonomic assignment of representative ASVs was carried out with the RDP (Ribosomal Database Project) classifier based on the Silva database (release: Silva 132) [33].

We used QIIME2 diversity analyses to compute alpha and beta diversity metrics. Alpha diversity is the ecological diversity within a sample. The following alpha diversity measures were calculated: Chao1 index (estimated microbial richness) [34], Faith’s phylogenetic diversity (Faith’s PD, richness index that additionally incorporates the phylogenetic relationships between microbial taxa) [35], and Shannon index (true biodiversity index not only taking into account microbial richness but also their evenness).

Beta diversity is a measure to compare differences in microbial community structure between samples. Aitchison and Bray–Curtis distances were calculated as quantitative beta diversity metrics (taking into account relative abundance profiles) [36,37], whereas the unweighted UniFrac distance was calculated as a qualitative measure of community dissimilarity (presence/absence of microbial taxa) that incorporates the phylogenetic relationships between ASVs [38].

To investigate potential similarities in the microbiota of humans and nonhumans, we determined the proportion of shared ASVs between all human subjects, as well as between nonhuman samples and humans in the cohort. In addition, we identified the shared ASVs that were classified as Enterobacteriaceae family in order to trace the similarities between humans and their living environments.

#### 2.2.3. Antimicrobial Resistance Genes

Metagenomic DNA of the samples included in the present study was previously subjected to real-time PCR using a 7900HT Fast Real-time PCR System (Applied Biosystem Inc., Foster City, CA, USA) to examine the presence and abundance of ARGs [26]. These data were used to correlate the presence of genes encoding extended-spectrum β-lactamases (ESBL) to specific ASVs within the Enterobacteriaceae family.

#### 2.2.4. Statistical Tests

Nonparametric two-sided Wilcoxon-rank sum and Kruskal–Wallis tests were used to test whether species richness (Chao1, Faith’s PD) and diversity (Shannon) were significantly different between sample types and within individuals in relation to geographical location. These analyses were also performed to determine the effect of antibiotic use on the human gut microbiota by comparing individuals who had and had not used antibiotics within the past 4 months. Wilcoxon tests also were used to compare the average of the shared ASVs and shared genera within individuals and within human and nonhuman samples in the same households versus other households.

To determine whether sample types significantly differed in microbial community structure, PERMANOVA (permutational multivariate analysis of variance) tests were applied using the beta-group significance command in QIIME2 [39]. In addition, we conducted these analyses to identify which variables mainly drive the structure of the human gut microbiota. A *p*-value (or false discovery rate (FDR) *q*-value) of less than 0.05 (two-sided) was considered statistically significant. To identify genera that significantly differed in relative abundance across sample groups, we used the Analysis of Composition of Microbiomes ANCOM v2.1 R package [40]. For declaring differentially abundant taxa, we set the cutoff value to 0.6 which referred to the W-statistic ≥110 for consideration of a significant difference. The association between antibiotic use and specific microbial taxa was adjusted for the following potential confounders: age, gender, and geographical clusters.

## 3. Results

### 3.1. Sample Types and Their Microbiome Sequence Information

The V4 hypervariable region of the 16S rRNA gene was sequenced for taxonomic analysis using QIIME2 to examine and compare the microbial compositions in human and animal feces, processed food, and water. After exclusion of samples with low sequencing depth (*n* = 4), 107 human fecal, 36 domestic animal fecal, 89 water, and 74 food samples were retained for downstream analysis. We obtained 3061 ASVs after clustering of 25,543,716 high-quality reads from 306 samples.

### 3.2. Microbiota of Human Gut in the Study Cohort

#### 3.2.1. Composition of Gut Microbiota of Individuals in the Context of Demographical Variables of Study Cohort

**Figure 1:**
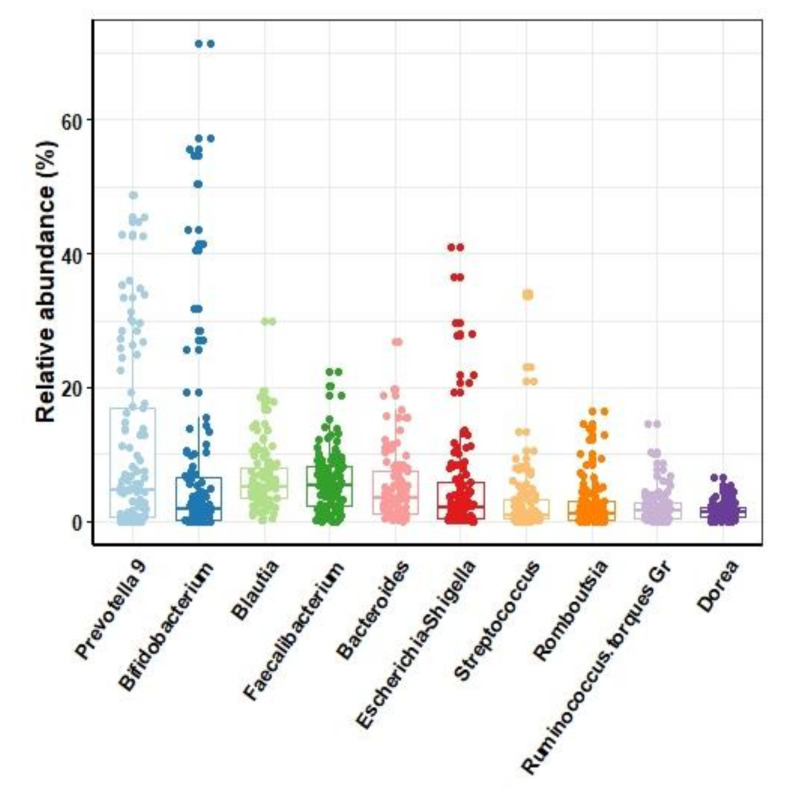
Relative abundance of the most predominant genera in human feces samples. Boxes show the median and interquartile ranges, while whiskers show the 95% ranges.

Overall, the human gut 16S rRNA gene sequences could be assigned to 13 phyla. The top four dominant phyla, accounting for 98.9% of all sequences, were Firmicutes (59.6%), Bacteroidetes (21.1%), Actinobacteria (9.3%), and Proteobacteria (8.8%). The most abundant genera (Figure 1) were *Faecalibacterium* with a median relative abundance (RA) of 5.4% (IQR 2.2–8.1), *Blautia* (5.2% (3.5–7.9)), *Prevotella* (5.1% (0.5–20.3)), and *Bifidobacterium* (1.76% (0.0–6.6)) (Figure 1).

**Figure 2:**
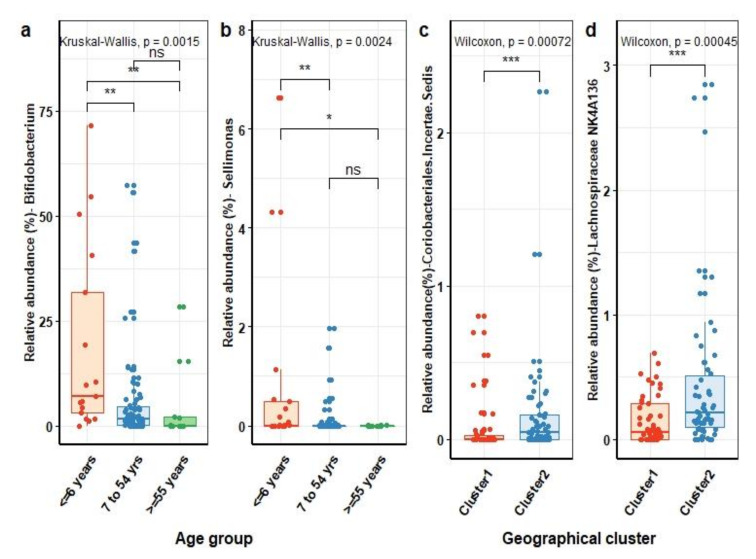
Human gut microbial genera in association with age and geographical location. Comparison of relative abundance (%) of *Bifidobacterium* (**a**) and *Sellimonas* (**b**) genera across age groups. Comparison of relative abundance (%) of *Coriobacteriales incertae sedis* (**c**) and *Lachnospiraceae NK4A136* (**d**) between individuals living in the two geographical clusters. Symbols show the statistical significance levels, ns indicates *p* > 5 × 10^−2^, * indicates *p* < 5 × 10^−2^, ** indicates *p* < 1 × 10^−2^, *** indicates *p* < 1 × 10^−3^.

To assess the compositional profile of the human gut microbiota, we compared the microbial richness, estimated by the Chao 1 index, and diversity, assessed by Shannon index, as well as the microbial community richness that incorporated phylogenetic relationship between ASVs (Faith’s PD) in relation to age, gender, and geographical location (see Supplementary Figures S2–S4, respectively). While there were no differences in fecal microbial richness and diversity between male and female individuals in our study cohort, the microbial diversity in children aged 6 years and below was lower than that of older children and adults. Both the estimated richness (Chao1) and the phylogenetic diversity (Faith’s PD) were significantly lower in the youngest age group as compared to individuals in the age between 7 and 55 years (two-sided Wilcoxon test, FDR *q* = 2 × 10^−2^ and *q* = 3 × 10^−2^, respectively). In line with this, the overall microbial composition of children aged 6 years or below was significantly different from other subjects (Bray–Curtis FDR *q* = 6 × 10^−3^, unweighted Unifrac, *q* = 1.5 × 10^−2^). The significant difference in microbial community structure between age groups was an indicator to investigate which genera were differentially abundant. Comparing the relative abundances at the genus level across three age groups (0–6 years (*n* = 17), 7–54 years (*n* = 79), and ≥55 years (*n* = 11)) (Figure 2a) without additional adjustment for other risk factors, we observed a gradual decrease in bifidobacteria with age (ANCOM, W = 161). In addition, the relative abundance of *Sellimonas* belonging to the Lachnospiraceae family in children aged 0–6 years was higher than in other age groups (ANCOM, W = 136) (Figure 2b). These observations withstood adjustment for antibiotic use and geographical region (ANCOM, W = 174 and W = 167, respectively).

We next examined the microbial composition of individuals in the context of living in geographically distinct sub-settings. Although the individuals lived in a narrow geographic area with similar living conditions and culture, we observed a significantly lower microbial richness and biodiversity (two-sided Wilcoxon test, FDR *q* = 1.4 × 10^−2^ and *q* = 9 × 10^−4^, respectively) among individuals living in cluster 1 (*n* = 49) as compared to individuals in clusters 2 (*n* = 58) (see Supplementary Figure S4). Comparing the microbial community structure of the human gut microbiota demonstrated quantitative differences between individuals living in cluster 1 and those living in cluster 2 (Bray–Curtis, FDR *q* = 3 × 10^−2^). Meanwhile, no differences in qualitative dissimilarities in microbial community structure were observed (unweighted Unifrac, FDR *q* = 1.35 × 10^−1^), indicating that the majority of species are not specific to the gut microbiota of individuals living in either one of these clusters but that some species may rather differ in their abundance between clusters. We indeed observed that the abundance of *Coriobacteriales incertae sedis* (ANCOM, W = 119) family and *Lachnospiraceae NK4A136* (ANCOM, W = 132) genus was significantly higher in the gut microbiota of individuals living in cluster 1 compared to individuals in cluster 2 (Figure 2c,d). These associations withstood adjusting for antibiotics use and age.

Examining the shared bacterial taxa between individuals in the same and in different geographical clusters showed that individuals living in cluster 2 shared more ASVs with each other than with individuals from cluster 1 (27.2% (20.3–32.8) versus 26.0% (19.5–31.3), *p* = 6.83 × 10^−5^). Individuals in cluster 1 on the other hand did not share more ASVs with each other than with individuals from the opposite cluster. Although the resolution of ASV-level data is insufficient to prove transmission of bacterial strains, this higher proportion of shared ASVs among individuals in cluster 2 might suggest that there is more dispersal within this cluster.

As dispersal of bacteria is likely most prominent within households, we next examined to what extent individuals within the same household share more bacterial taxa than individuals from different households. These results confirmed that the proportion of shared bacterial taxa was significantly higher among individuals from the same household (median 27.9% (21.3–34.1) as compared to individuals from different households (26.2% (19.5–31.6), Wilcoxon *p* = 8.05 × 10^−3^). When stratifying these analyses for age, both children under the age of 6 years and individuals above the age of 6 years shared more bacterial taxa when living in the same household (see Supplementary Table S3). These results, thus, confirm the sharing of bacteria among household members.

#### 3.2.2. Composition of Gut Microbiome of Healthy People in the Context of Their Antibiotic Use History

**Figure 3:**
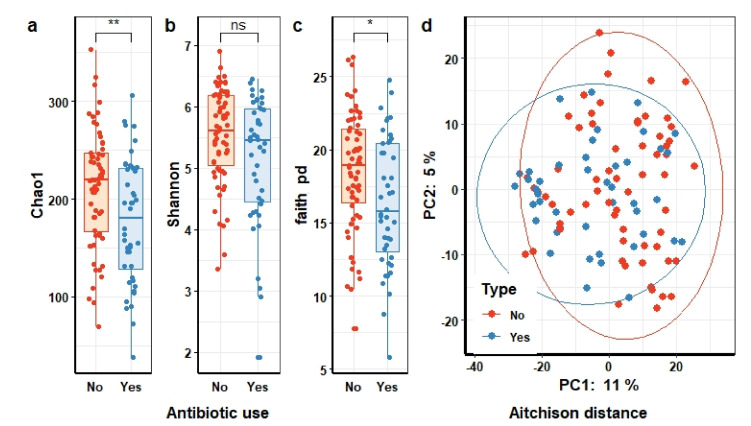
Differences in gut microbial community structures and taxonomic composition in association to antibiotic use in the 4 months prior to feces sampling. Differences in microbial richness (Chao1) (**a**) and diversity (Shannon (**b**) and Faith’s pd (**c**)) between people who did or did not use antibiotics. Statistical significance (FDR *q*-value < 5 × 10^−2^) determined by Wilcoxon test. (**d**) Principal component analysis based on Aitchison distance colored according to antibiotic use (PERMANOVA test, FDR *q*-value < 5 × 10^−2^). Each point represents the gut microbial community structure of an individual that did (blue) or did not (red) use antibiotics. Symbols show the statistical significance levels, ns indicates *p* > 5 × 10^−2^, * indicates *p* < 5 × 10^−2^, ** indicates *p* < 1 × 10^−2^.

Among all 107 participants, 44 individuals used antibiotics at least once during the past 4 months. Like previous findings about the influence of antibiotic use on microbial compositions, we observed a reduction in gut microbial richness and diversity among individuals who used antibiotics (*n* = 44) as compared to individuals who did not (*n* = 63) use antibiotics in the past 4 months (Figure 3a). Both the estimated richness (Chao1) (Figure 3a) and the phylogenetic diversity (Faith’s PD) (Figure 3c) were significantly lower in individuals who used antibiotics (two-sided Wilcoxon test, FDR *q* = 7 × 10^−3^ and *q* = 1.7 × 10^−2^, respectively), whereas the microbial diversity as measured by the Shannon index did not reach statistical significance (two-sided Wilcoxon test, *q* = 8 × 10^−2^) (Figure 3b). In addition, Aitchison distance showed significant differences in microbial community structure in association with antibiotic use (PERMANOVA, FDR *q* = 9 × 10^−3^). The results were confirmed when using other beta diversity indices that measure not only the dissimilarities between microbial composition but also incorporate phylogenetic relationships (unweighted Unifrac, FDR *q* = 1.6 × 10^−2^). However, no clear visual separation in the microbial community structure of individuals with or without recent antibiotic use could be observed when visualizing these dissimilarities using principal component analysis (PCA). This indicates that although recent antibiotic use significantly influenced the microbial community structure, it did not result in profound shifts in the ordination plot along the first two components (Figure 3d). It should, however, be noted that the first two components only explained 11% and 5% of the overall variance, respectively, indicating relatively poor representation of the high-dimensional data.

We next investigated which specific taxa were perturbed due to antibiotic use in our cohort. Unlike previous studies, we observed only few genera, belonging to the Clostridiales order, to be significantly associated with antibiotic use (two-sided Wilcoxon test *q* < 5 × 10^−2^, antibiotic use variable without adjusting for age and geographic variables), including *Lachnospiraceae FCS020* group, and *Ruminococcus gnavus* group genera. The relative abundance of *Lachnospiraceae FCS020* group in individuals who used antibiotics in the past 4 months was significantly lower than in individuals without antibiotics use (ANCOM, W = 124) and this difference withstood adjustment for age and geographic variables. When adjusting for either age or geographic variables the difference in relative abundance of the *Ruminococcus gnavus* group was no longer statistically significant, but we did observe a slight decrease in relative abundance of *Ruminococcaceae UCG-002* in individuals who used antibiotics in the past 4 months as compared to individuals without antibiotic use (ANCOM, W = 120) (see Supplementary Table S4).

### 3.3. Microbiota of Nonhuman Samples

#### 3.3.1. The Microbial Composition of Domestic Animals

To perform in-depth characterization of the nonhuman microbiomes in the present community, we investigated the differences in composition between sample types (i.e., animal feces, food, and water), as well as between samples from the same origin but from the different geographic clusters.

**Figure 4:**
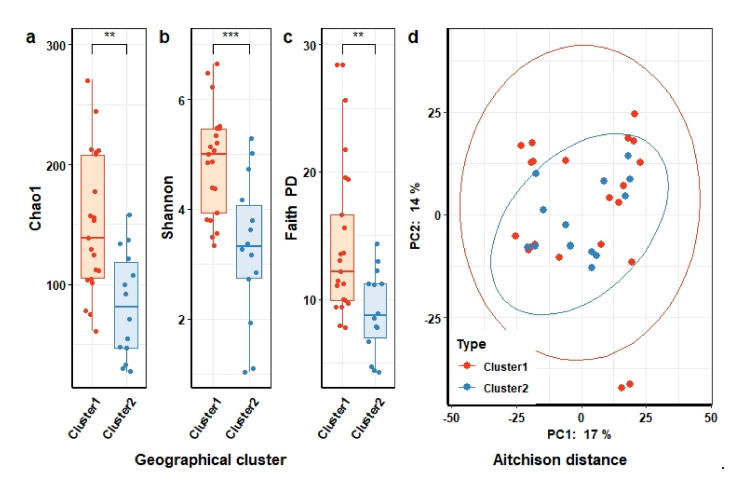
Microbial richness, diversity, and community structure of domestic animal gut microbiota (both chickens and dogs) living in different geographical clusters. Differences in the microbial richness (Chao1 (**a**)) and diversity (Shannon (**b**) and Faith’s PD (**c**)) between animals living in cluster 1 versus animals living in cluster 2 compared by Wilcoxon test. Difference is significant at FDR *q*-value < 5 × 10^−2^. (**d**) Ordination of the gut microbiota community structures of geographical cluster based on principal component analysis of Aitchison distance. Each point represents the gut microbial community structure of an individual animal and is colored according to geographic cluster with animals from cluster 1 colored in red and animals from cluster 2 colored in blue. (PERMANOVA test, FDR *q*-value ≥ 5 × 10^−2^ for geographic cluster). Symbols show the statistical significance levels, ** indicates *p* < 1 × 10^−2^, *** indicates *p* < 1 × 10^−3^.

Interestingly, we neither observed differences in microbial richness and biodiversity nor in microbial community structure between the two domestic animal species, chicken (*n* = 11) and dogs (*n* = 24) (see Supplementary Table S5). When comparing alpha diversity values between animals living in different geographical clusters, the microbial richness and biodiversity of animals in cluster 1 (*n* = 21) was significantly higher than in cluster 2 (*n* = 14) (two-sided Wilcoxon test; FDR *q* < 5 × 10^−2^) (Figure 4a–c). Although we observed no dissimilarities between cluster 1 and cluster 2 in measures of microbial community structure (PREMANOVA, FDR *q* > 5 × 10^−2^) (Figure 4d), the qualitative dissimilarity, incorporating phylogenetic relationships, appeared significantly different (unweighted UniFrac distance, PERMANOVA FDR *q* = 2.9 × 10^−2^; see Supplementary Table S5). These findings indicate that the microbial community structure of feces from domestic animals was associated with the geographical location and could be related to the contamination from soil, as we could not collect the fresh feces from domestic animals.

Interestingly, we neither observed differences in microbial richness and biodiversity nor in microbial community structure between the two domestic animal species, chicken (*n* = 11) and dogs (*n* = 24) (see Supplementary Table S5). When comparing alpha diversity values between animals living in different geographical clusters, the microbial richness and biodiversity of animals in cluster 1 (*n* = 21) was significantly higher than in cluster 2 (*n* = 14) (two-sided Wilcoxon test; FDR *q* < 5 × 10^−2^) (Figure 4a–c). Although we observed no dissimilarities between cluster 1 and cluster 2 in measures of microbial community structure (PREMANOVA, FDR *q* > 5 × 10^−2^) (Figure 4d), the qualitative dissimilarity, incorporating phylogenetic relationships, appeared significantly different (unweighted UniFrac distance, PERMANOVA FDR *q* = 2.9 × 10^−2^; see Supplementary Table S5). These findings indicate that the microbial community structure of feces from domestic animals was associated with the geographical location and could be related to the contamination from soil, as we could not collect the fresh feces from domestic animals.

We also investigated which microorganisms were dominant and more abundant in different animal species. At the genus level, 181 genera, accounting for 98.3% of total sequences, were detected across all samples from domestic animals. The most dominant genera were *Escherichia* with a median of relative abundance (RA) of 7.1% (2.0–15.6)), *Lactobacillus* (median 5.5% (0.5–18.8)), *Acinetobacter* (median 3.2% (0–10)), and *Clostridium sensu stricto* (median 2.3% (0.5–6.0)). Significant differences in relative abundance at genus level were, however, neither observed when comparing different animal species nor when comparing animals from the different geographic clusters. These latter results should, however, be interpreted with caution due to the potential lack of statistical power related to the relatively small sample size.

#### 3.3.2. The Microbial Composition of Water

**Figure 5:**
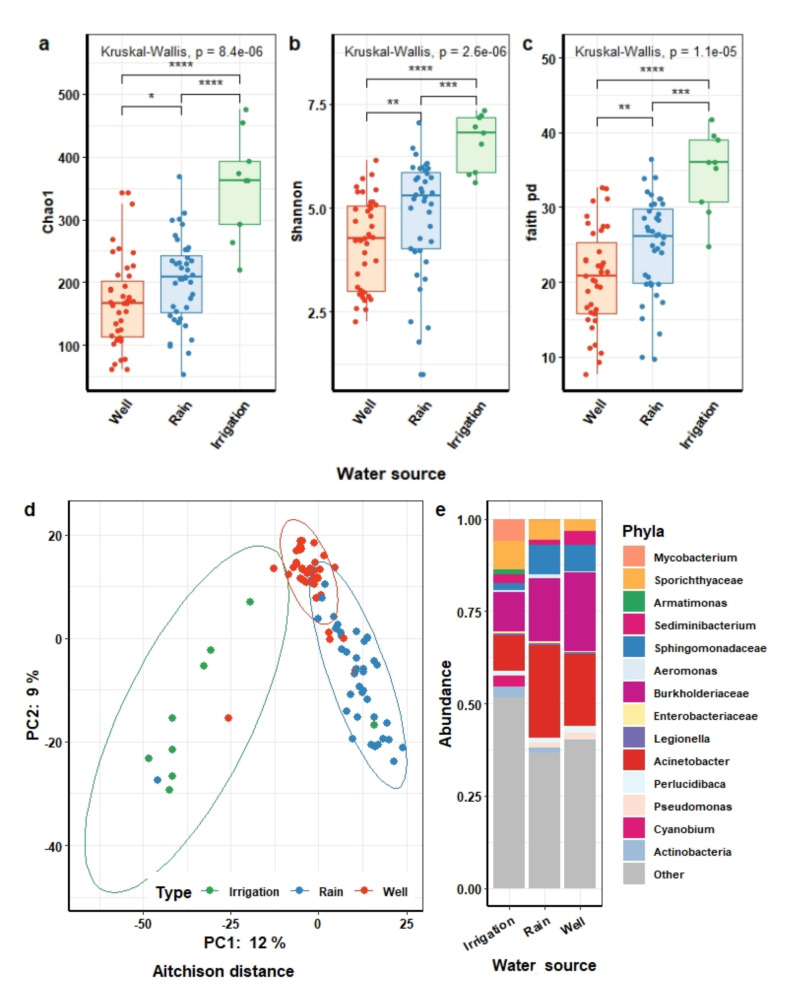
Microbial richness, diversity, community structure, and dominant taxa of different water sources. Differences in microbial richness (Chao1) (**a**) and diversity (Shannon (**b**) and Faith’s PD (**c**)) of different water sources compared by Wilcoxon tests. Difference is significant at FDR *q*-value < 5 × 10^−2^. (**d**) Ordination of the microbiota community structures of water samples based on principal component analysis of Aitchison distance (PERMANOVA test, FDR *q*-value < 5 × 10^−2^). Each point represents the microbiota community structure of well (red), irrigation water (green), and rainwater (blue), respectively. (**e**) The relative abundance of major bacterial genera in different water sources. Symbols show the statistical significance levels, ns indicates *p* ≥ 5 × 10^−2^, * indicates *p* < 5 × 10^−2^, ** indicates *p* < 1 × 10^−2^, *** indicates *p* < 1 × 10^−3^, **** indicates *p* < 1 × 10^−4^.

Unlike the findings in domestic animals, water in different sources has different compositions. The estimated microbial richness (Chao1), Shannon index, and phylogenetic diversity (Faith’s PD) all significantly differed between types of water, with irrigation water being the most diverse, followed by rainwater and water from wells (two-sided Wilcoxon singed-rank test; FDR *q* < 5 × 10^−2^) (Figure 5a–c). PCA demonstrated that the microbial composition of irrigation water was profoundly different from the composition of the other two water sources (Aitchison, PERMANOVA, FDR *q* = 1 × 10^−3^) (Figure 5d), and this held true for other indices of microbial community structure (Bray–Cutis, unweighted UniFrac, PERMANOVA, FDR *q* = 1 × 10^−3^) (see Supplementary Table S6).

We identified 28 phyla in water samples from three sources, which is far greater than the 13 and 17 phyla observed in humans and animals, respectively. At a lower taxonomic level, among the most prevalent 349 genera, which were detected in at least 10% of all water samples, accounting for 98.1% of total sequences, there were 184 uncultivable or unidentified genera, accounting for 41% of total sequences. The genus *Acinetobacter* and the family Burkholderiaceae were the most abundant bacteria with a median RA of 5.4% (0.5–38.4) and 6.6% (3.2–16.4), respectively. *Novosphingobium*, *Sediminibacterium*, *Pseudomonas*, and *Aquabacterium* genera were detected at low abundance (ranging in median RA from 0.2% (0.1–1.1) to 0.9% (0.2–4.2)), but each of these genera was highly prevalent and detected in more than 90% of all water samples (Figure 5e). The difference in microbial community structure between water sources was also evident when examining the relative abundance of specific microbial taxa. Overall, 116 genera differed in abundance across the water samples from different sources (ANCOM, W > 231), see Supplementary Table S7). These differences strongly supported that the water’s source was the key factor responsible for the distinction and unique compositional profiles between water samples.

#### 3.3.3. The Microbial Composition of Processed Food

In this study, we also investigated the microbiota of cooked food including food that individuals ate as part of their daily meal at a single timepoint. We observed that the top four phyla, identified in at least 83.8% of food samples, were Firmicutes (75/75), Proteobacteria (74/75), Bacteroidetes (63/75), and Actinobacteria (62/75). The low biomass and microbial composition of the processed food sampled at a single timepoint is likely not representative for the overall microbiota related to the food consumed by the participants in our study cohort. Therefore, we only used these data as an additional nonhuman microbiota in order to investigate the potential dispersal of taxa between individuals and their environmental factors.

### 3.4. Human versus Nonhuman Microbiota

#### 3.4.1. Overall Composition and Diversity of the Human Gut Microbiota in Relation to Microbial Community of Domestic Animal Gut, Water, and Processed Food

We explored the microbial composition of the entire cohort and visualized the shared microbial taxa between humans and nonhuman samples. The microbial richness and diversity of human fecal samples was significantly higher as compared to feces from animals and food samples, but not of water from different sources, in our cohort. Both estimated richness (Chao1) and Shannon diversity were extremely high in human as compared to animal and food samples (two-sided Wilcoxon, FDR-adjusted *p*-values (*q*) = 1.472 × 10^−7^ and *q* = 4.421 × 10^−6^, respectively, for humans versus animals, and FDR-adjusted *p*-value (*q*) = 5.343 × 10^−25^ and *q* = 3.051 × 10^−^^24^, respectively, for humans versus food).

**Figure 6:**
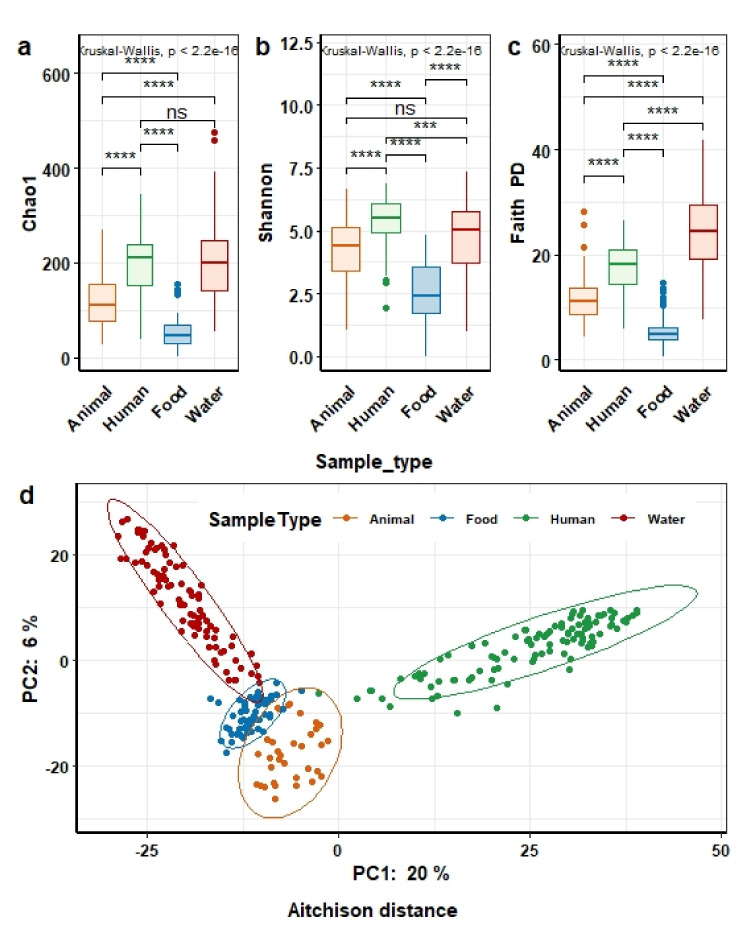
Microbial composition of human and animal gut, water, and food. Differences in estimated microbial richness (Chao1) (**a**) and diversity (Shannon (**b**) and Faith’s PD (**c**)) across sample types. Kruskal–Wallis test followed by Wilcoxon test for post hoc comparisons of four sample types (humans, animals, food, and water). *** *p* < 1 × 10^−3^, **** *p* < 1 ×10^−4^. (**d**) Principal component analysis (PCA) based on Aitchison distance. Each point represents the microbial community structure of an individual animal stool (orange), human stool (green), food (dark blue), and water (red) sample. (PERMANOVA test, *p* value < 1 × 10^−3^).

In addition, results were similar when assessing community richness by including phylogenetic relationships between ASVs using Faith’s PD. Despite no significant difference in estimated richness (FDR *q* = 7.5 × 10^−1^) between human and water (Figure 6a), the Shannon diversity, which incorporates both abundance and evenness of taxa, and the phylogenetic diversity (Figure 6b,c) remained significantly higher in human as compared to water samples (two-sided Wilcoxon, FDR *q* = 5.56 × 10^−4^, *q* = 1.14 × 10^−9^, respectively). In line with significant differences in alpha diversity, PCA demonstrated that human gut microbiota clustered apart from domestic animals, water, and food along the first component. Meanwhile, domestic animals, water, and food were relatively more similar to each other (Figure 6d).

The microbial community composition of the human and animal gut, processed food, and water was assigned to 31 bacterial phyla. The top seven dominant phyla, accounting for 98.7% of the total sequences, were Firmicutes (38.8%), Proteobacteria (38.7%), Bacteroidetes (11.9%), Actinobacteria (6.7%), Planctomycetes (1.1%), Verrucomicrobia (0.9%), and Fusobacteria (0.3%). The Firmicutes phylum was predominant in the human (median relative abundance 62.0% (46.5–73.3%)) and animal (median 53.2% (39.4–74.3%)) gut and food (median 47.8% (11.8–91.6%)). Meanwhile, Proteobacteria was the dominant phylum in water (median 72% (58.0–87.0)), while also being highly abundant in food (median 47.7% (7.2–85.0)) and in animal stools (median 29.4% (6.3–44.3)). The Verrucomicrobia phylum was low abundant in all sample types with a median relative abundance, ranging from 0% (0–0.001) in feces from human to 0.9% (0.1–3.5) in water; however, this phylum was much more prevalent in water samples (96%, 87/91) than in human feces (32.7%, 35/107).

**Figure 7:**
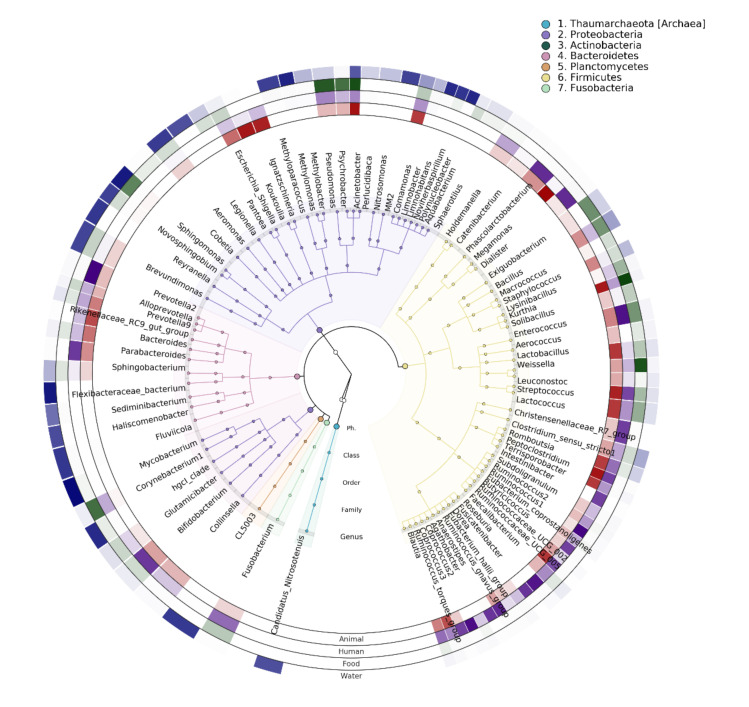
Cladogram depicting the relative abundance of the bacterial genera detected in the different sample types. Only genera with a mean relative abundance of >0.5% in at least one of the sample types were included. Background and branch colors reflect the different phyla. The color density of the four outer rings reflects the relative abundance (arcsine square root transformed) of the genera in the different sample types (with opaque color indicating a relative abundance of 1 and fully transparent indicating a relative abundance of 0).

At lower taxonomic levels, 524 genera, accounting for 99.3% of total sequences, were detected across 306 samples. The genera *Acinetobacter*, *Prevotella*, *Staphylococcus*, and genera belonging to family Enterobacteriaceae were the most abundant, accounting for 27.2% of all sequences. Many genera were presented in all four types of samples such as *Streptococcus*, *Bacillus*, *Comamonas*, *Lactococcus*, and *Acinetobacter* (Figure 7). Meanwhile, some other genera were only prevalent in specific sample types such as *Mycobacterium* and *Fluviicola* in water, as well as *Intestinibacter* and *Roseburia* in human gut. Notably, our study showed that many genera belonging to Proteobacteria and Actinobacteria were detected in both animal gut and water but absent or sporadically detected in the human gut.

#### 3.4.2. Potential Dispersal of Microbial Taxa between the Microbiota of Humans and Nonhumans

Although the depth of strain level cannot be determined with 16S rRNA gene amplicon data, amplicon sequence variants give a much better resolution than clustering into operational taxonomic units. We, therefore, explored the potential dispersal of ASVs between microbial ecosystems. We observed that humans shared on average 26.2% (19.6–31.6) ASVs in their feces with other individuals. When comparing human feces with other microbial habitats, we observed that feces from humans shared a median of 7.44% (3.2–9.9) of its ASVs with the feces from domestic animals, 2.2% (1.2–2.8) with water, and 3.1% (1.5–4.1) with processed food. The proportion of shared ASVs between human and nonhuman samples from the same HH was, however, not significantly higher than the proportion of shared ASVs between those samples from different HHs (see Supplementary Figure S5). The most commonly shared genera between humans and nonhumans belonged to the Enterobacteriaceae family.

In order to investigate specific ASVs within genera that are widely shared between humans and their living environment, we subsequently focused on those ASVs classified as Enterobacteriaceae. We observed that 50 ASVs classified as Enterobacteriaceae across sample types. Each individual shared at least one Enterobacteriaceae ASV with animals, water, and food samples; however, the proportion of these shared ASVs between human and nonhuman samples did not significantly differ between samples from the same HHs as compared to samples from different HHs (Wilcoxon, *p*-value > 5 × 10^−2^) (see Supplementary Figure S6). Interestingly, when looking at the ASVs shared between different hosts and associated with specific ARGs, we observed several ASVs that were positively correlated with the presence of ARGs in both human and animal samples, suggesting exchange of antimicrobial-resistant strains between human and animal reservoirs. In particular, we observed that an ASV belonging to the Enterobacteriaceae family, present in 46.7% (50/107) of human samples and 28.6% (10/35) of animal samples, was positively correlated with the presence of *bla_ctx-m-2_* in fecal samples. In 30.0% (15/50) of humans carrying this ASV and 40% (4/10) of animals carrying this ASV, we also detected *bla_ctx-m-2_* (*p* = 1.6 × 10^−3^ and *p* = 2.6 × 10^−2^, respectively).

## 4. Discussion

In this study, we characterized the microbiota of the human and domestic animal gut and their associated environments using 16S rRNA gene sequencing in a community setting with a high frequency of antibiotic consumption. We described the core group of bacterial taxa in feces from humans and domestic animals, food, and water from three different sources. The results of our study indicated that antibiotic use decreased the microbial diversity in the human gut, although only few bacterial taxa appeared to be affected in the present population. We furthermore showed that, even in the confined habitat of seven neighboring villages, the microbiota composition of both humans and animals appeared to be associated to geographic location. Lastly, the human gut microbial composition differed most from all other sample types analyzed in the present study, and the limited number of shared ASVs between habitats indicates the presence of barriers for successful microbial colonization between ecosystems. Such barriers are most likely attributable to environmental filtering or niche-based interactions in various ecosystems, including the human gut. Host-related factors such as body temperature or endogenous food substrates (e.g., glycoproteins, bile salts) or exogenous factors such as dietary food substrates can select for the growth of certain bacteria while preventing the growth of others. Such environmental selection can be in the form of a habitat filter in which the host influences bacterial colonization but not vice versa (e.g., body temperature) or in the form of a bidirectional host–microbe interaction (e.g., the interaction between bacteria and the host’s immune system). In addition to this environmental filtering, niche-limitation can form a colonization barrier. In this process, the closely related bacterial species or strains already present in an ecosystem prevent the colonization of related taxa.

In the current study, we focused on describing the patterns of human gut microbiota in a cohort with a high level of antibiotic use. Our results showed that the *Prevotella*, *Bifidobacterium*, *Blautia*, *Faecalibacterium*, and *Bacteroides* genera were the most abundant among rural individuals in Vietnam. The median relative abundance of *Bifidobacterium* in our population was 1.76% (0–6.6) which is similar to the estimated abundance of *Bifidobacterium* of below 2% as described in US, South Korea, and Europe [41,42,43]. The *Bifidobacterium* genus is known to confer health benefits through metabolic activities such as involving in oligosaccharide fermentation [44], and a reduction in its relative abundance in human adult gut composition is an indicator of antibiotic use such as amoxicillin, ampicillin, and gentamicin, as described previously [45,46]. Interestingly, we observed a higher abundance of the Proteobacteria phylum (median 3.5% (1.3–9.0)) when compared to Europeans, Americans, and Asians in other areas (less than 2%) [43]. In particular, it was much higher than that of individuals living in other traditional populations with low antibiotic exposure such as the Hadza hunter-gatherers of Tazania or the Yanomami in the Amazon jungle [47,48]. A study of infant gut found that the Enterobacteriaceae family consists mostly of opportunistic pathogens with a high level of antibiotic resistance genes [49]. Moreover, a previous study showed that there was an acute blooming of *Klebsiella* spp. and *E. coli* in the gut microbiota after antibiotic treatment [13]. The relatively high abundance of Proteobacteria in the human gut microbiota in our cohort is likely the result of intensive exposure to antibiotics.

Our results are in agreement with recent studies indicating the significant relationship between age groups and the abundance of particular genera [42,50]. For instance, our finding was consistent with previous reports of the decreasing abundance of bifidobacteria with age [50,51]. The *Sellimonas* genus was also found to decrease with age but there is limited evidence on its critical role in the gut microbiota so far [52]. In this study, we indicated the difference in the relative abundance of the *Lachnospiraceae NK4A136* genus between individuals living in two geographical locations. In previous reports, the low abundance of this genus, which belongs to short-chain fatty-acid-producing bacteria, was associated with obesity in humans and mice [53,54,55]. We only recorded that the households living in cluster 1 consumed meat more frequently than the households living in cluster 2 (more than once versus one time per week, chi-square, *p*-value < 0.05), but we had no information to link the abundance of this genus to the body mass index of individuals.

In contrast to the majority of previous studies [13,56], we observed only a modest difference in microbial composition between individuals who used and who did not use antibiotics in the previous 4 months. Previous studies reported that, after short-term antibiotic use, the diversity of the gut microbiome is dramatically reduced and needs several months to recover to its original state. Some other studies demonstrated that many common species in the gut microbiome of healthy adults disappeared up to 180 days after antibiotic treatment [13,57]. Additionally, we only observed that the abundance of the *Lachnospiraceae FCS020* group was significantly reduced among individuals who recently consumed antibiotics. Our results might suggest that frequent exposure to antibiotics in the entire population might have resulted in a new steady state of microbial composition that is less influenced by subsequent antibiotic pressure [56,58]. This slight impact might also be explained by the fact that beta-lactam antibiotics, whose perturbations on the microbiome are less profound than macrolides or quinolones, were the most commonly used in our study [59].

Our results also highlighted the similarities and dissimilarities in the microbial community structure between human and nonhuman samples. We observed that human gut had the highest diversity followed by water, domestic animals, and cooked food by considering the estimate richness and biodiversity indices. Firmicutes and Bacteroidetes consist of major genera which play critical roles in forming the gut microbial composition of humans and animals [50,60]. In contrast, the phylum Proteobacteria, which includes the genera *Pseudomonas* and *Acinetobacter*, and the family Enterobacteriaceae, is the most predominant in water including freshwater, sewage, and river [61,62]. Although the microbial compositions of humans, animals, and water are unique, our results showed a small overlap at the ASV level. In the same habitat, human gut microbiota shared nearly 8% of their ASVs with domestic animal gut and 4% with water. Most of the shared ASVs between human and nonhuman samples were classified as Enterobacteriaceae family, which is a potential reservoir for shedding antibiotic resistance genes. Recent studies suggest that, through feces, humans and domestic animals disseminate antibiotic-resistant bacteria into the environment [63]. Our previous report showed that antibiotic resistance genes were detected in water at a low proportion compared to humans and animals [26]. Along with the antibiotic pollution in water resources, antibiotic-resistant microbes might become a part of water microbiota [64]. Therefore, a possible consequence of the high pressure of exposure to antibiotics is that humans may become more receptive to be colonized with resistant bacteria from their environment.

The present study had several limitations. Due to the cross-sectional nature of the sample collection, we were not able to longitudinally follow the microbiota recovery after antibiotic exposure. Additionally, the 16S rRNA gene amplification-based sequencing approach did not allow us to directly match the antibiotic resistance genes (ARGs) with their carrier taxa. Using sequence-based metagenomics, a recent study linked ARGs with the 50 most abundant genera in urban sewage from 60 countries [61], while another report also showed that most of the ARG-carrying sequences in migratory birds originate from Proteobacteria [62]. Future studies collecting repeated samples at different timepoints are needed to further extend our knowledge on the long-term impact of antibiotic use on microbial ecosystems in low- and middle-resource settings.

## 5. Conclusions

In conclusion, we characterized the microbiota of feces from humans, domestic animals, and their direct environment in a Vietnamese community with high antibiotic use. Our results are in agreement with previous studies about the transition points in gut microbial composition with age, as well as indicate some microbial patterns related to geographical location. In addition, the small difference between antibiotic users suggested that exposure to antibiotics might not play an important role in microbial perturbation in a cohort with a high background of antibiotic use. We provide proof of taxa that could be potential ARGs carriers and might spread to the direct environment of humans through their interaction with the human gut microbiota. Indeed, we found a potential exchange of the *bla_ctx-m-2_* gene, which was one of the most prevalent ESBLs between feces from humans and animals in this study.

## Data Availability

The data for this study have been deposited in the European Nucleotide Archive (ENA) at EMBL-EBI under accession number PRJEB47865 (https://www.ebi.ac.uk/ena/browser/view/ (accessed on 5 October 2021) PRJEB47865). Accession Numbers have been listed in Supplementary Table S8: Accession Number sheet.

## Supplementary Materials

The following are available online at https://www.mdpi.com/article/10.3390/microorganisms9102115/s1, Figure S1: Geographical location of households, Figure S2: Comparison of the microbial richness and diversity in relation to age, Figure S3: Comparison of the microbial richness and diversity in relation to gender, Figure S4: Comparison of the microbial richness and diversity in relation to geographical cluster (based on the location of households), Figure S5: Comparison of the shared ASVs between humans and between human and non-human samples from the same and from different households (HH), Figure S6: Number of ASVs classified as *Enterobacteriaceae* in feces from humans, domestic animals, water and food, Figure S7: Comparison of microbial richness and diversity in children under 6 years living in different geographical clusters, Figure S8: Sample-based rarefaction curves, Table S1: Demographical characteristics of the cohort, Table S2: Characteristics of the geographical clusters, Table S3: Statistical comparison of shared ASVs between age groups and between geographical clusters, Table S4: Differential abundance of microbial taxa across stool samples from humans of different age groups and geographical clusters, Table S5: Alpha and beta diversity of domestic animal stool sample, Table S6: Alpha and beta diversity of water samples from different sources, Table S7: Differential abundance of microbial taxa across water samples from different sources, Table S8: Accession Number sheet.

## Abbreviations

ASVs: amplicon sequencing variables, ARG: antibiotic resistance genes, DNA: deoxyribonucleic acid, RNA: ribonucleic acid.
