# Supplementary material for: Cross-Sectional Analysis of the Microbiota of Human Gut and Its Direct Environment in a Household Cohort with High Background of Antibiotic Use
Source: Microorganisms. 2021 Oct 8;9(10):2115. doi: 10.3390/microorganisms9102115 (PMC8539590; doi:10.3390/microorganisms9102115)
Supplement: Supplementary file 1 [file microorganisms-09-02115-s001.zip › Supplementary_Figure_legends.pdf]

Cross-Sectional Analysis of the Microbiota of Human Gut and Its Direct Environment in a Household Cohort with High Background of Antibiotic Use

**Bich Vu Thi Ngoc [1,2], Hai Ho Bich [1], Gianluca Galazzo [3],Dung Vu Tien Viet [1], Melissa Oomen [3], Trang Nghiem Nguyen Minh [1], Hoang Tran Huy[4], Hindrik Rogier van Doorn [1,5], Heiman F.L., Wertheim [2,5] and
John Penders [3,*]**

Supplementary Figures

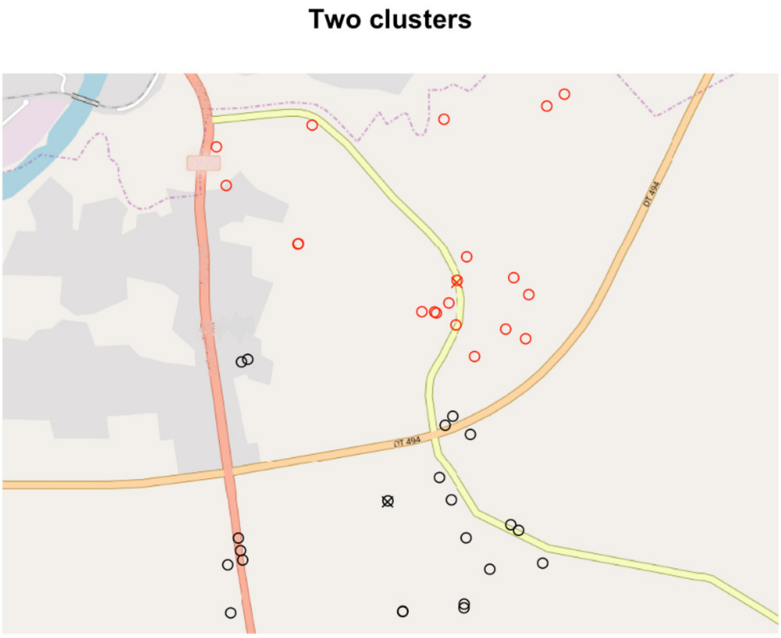

Figure S1. Geographical location of households. Each spot represents the location of a household in the community. Red and black spots indicate the households belonging to geographical cluster 1, and cluster 2, respectively. Dark yellow line indicates the provincial road, light yellow line indicates the river enclosing the study cohort.

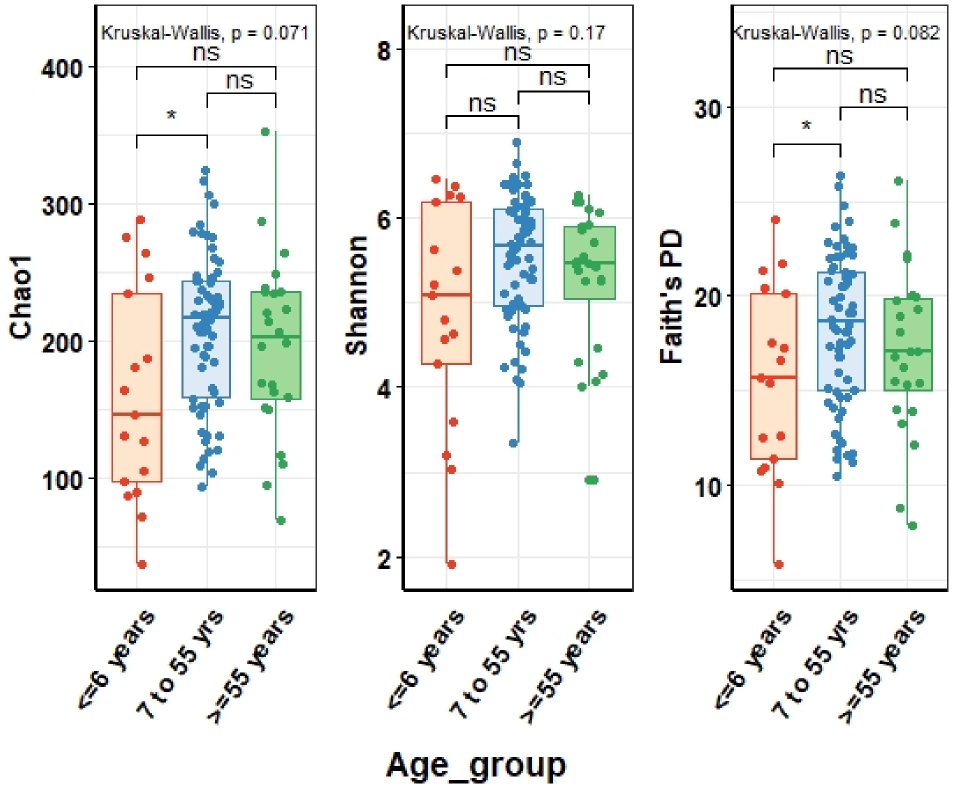

Figure S2: Comparison of the microbial richness (Chao 1), bio-diversity (Shannon index) and microbial diversity incorporating the phylogenetic relationship between ASVs (Faith's PD) in relation to age groups.

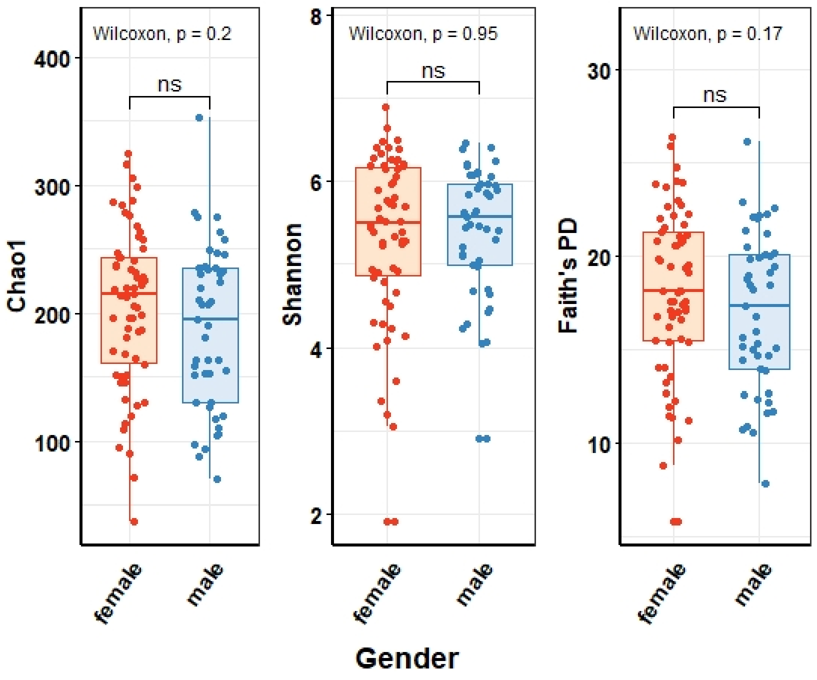

Figure S3: Comparison of the microbial richness (Chao 1), bio-diversity (Shannon index) and microbial diversity incorporating the phylogenetic relationship between ASVs (Faith's PD) in relation to gender.

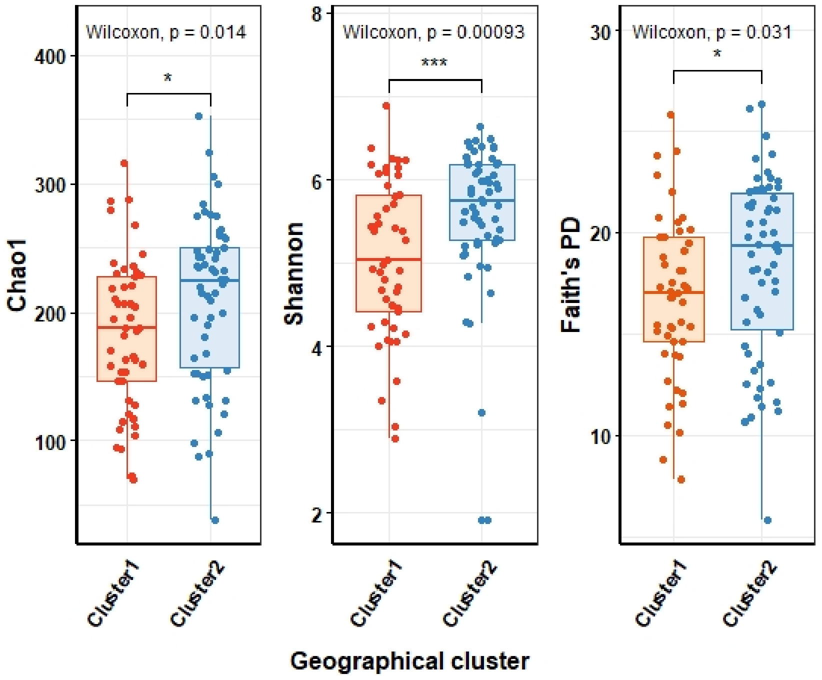

Figure S4: Comparison of the microbial richness (Chao 1), bio-diversity (Shannon index) and microbial diversity incorporating the phylogenetic relationship between ASVs (Faith's PD) in relation to the geographical cluster in which study subjects resided.

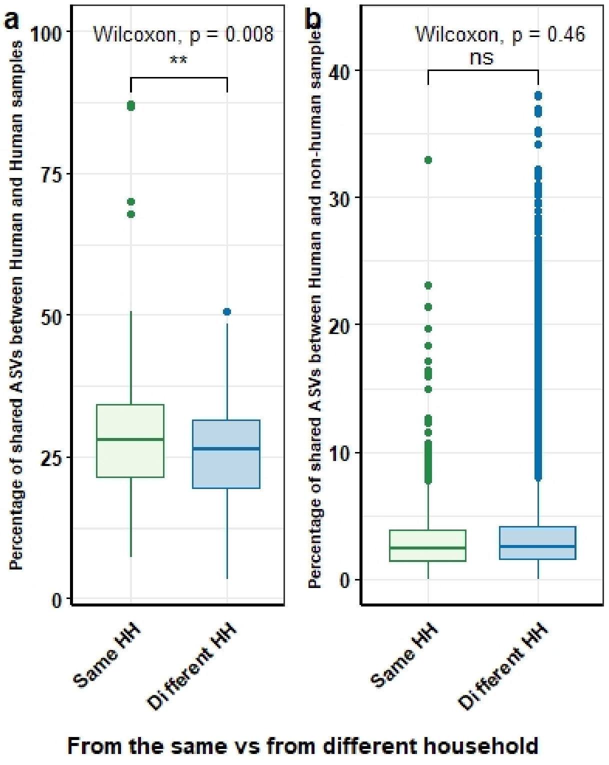

Figure S5: Comparison of the shared ASVs between humans and between human and non-human samples (Wilcoxon test) from the same and from different households (HH)

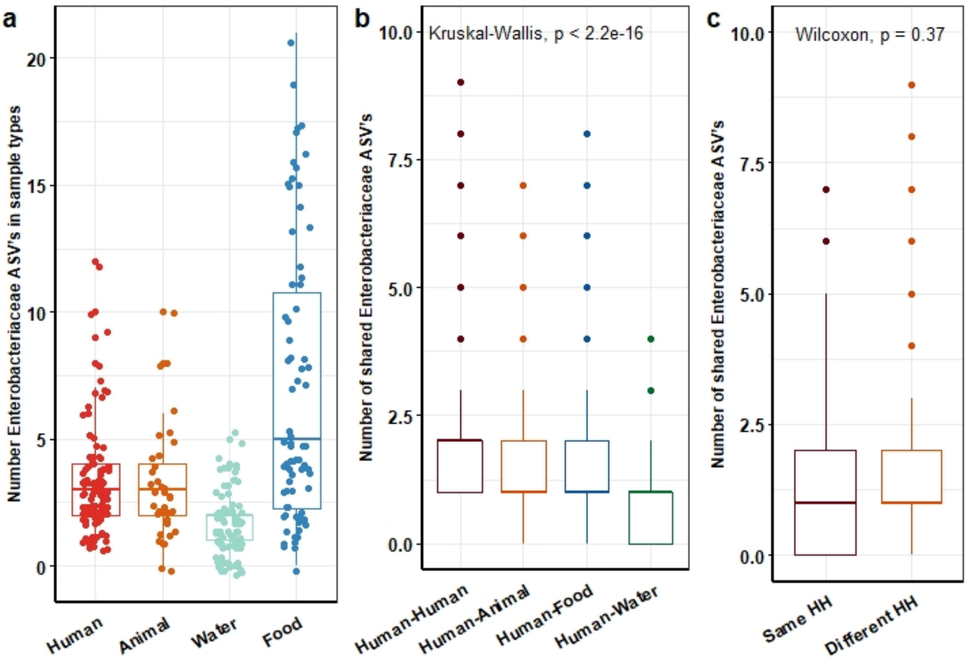

Figure S6: Number of ASVs classified as *Enterobacteriacea* in feces from humans, domestic animals, water and food (a). Number of shared ASVs classified as *Enterobacteriacea* between human subjects and between human versus other sample types (b), comparisons of shared ASVs

within the *Enterobacteriacea* genus between human samples and between human and non-human samples from the same and different households (HH) (c).

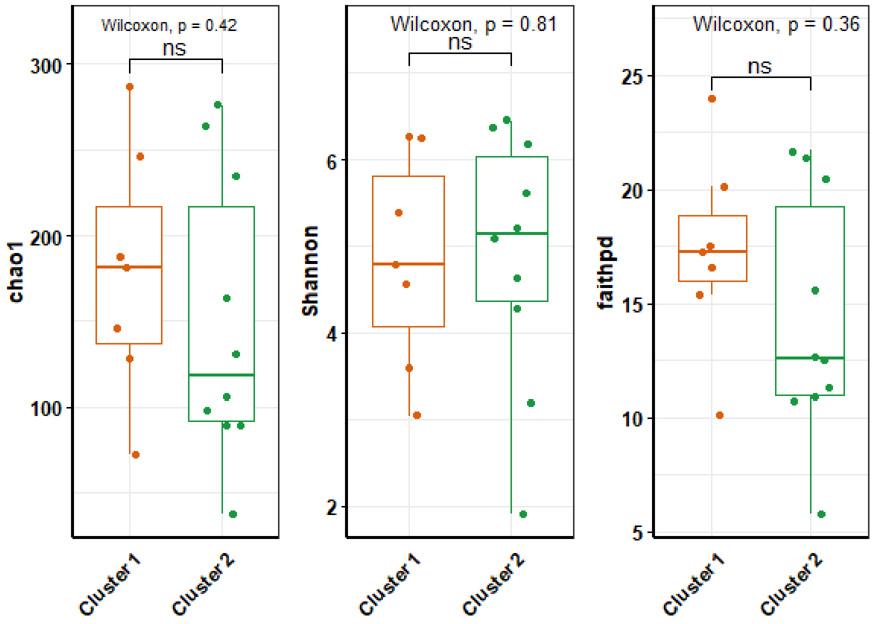

Figure S7: Comparison of microbial richness (Chao 1), bio-diversity (Shannon index) and the microbial diversity incorporating the phylogenetic relationship between ASVs (Faith's PD) between children under 6 years living in different geographical clusters.

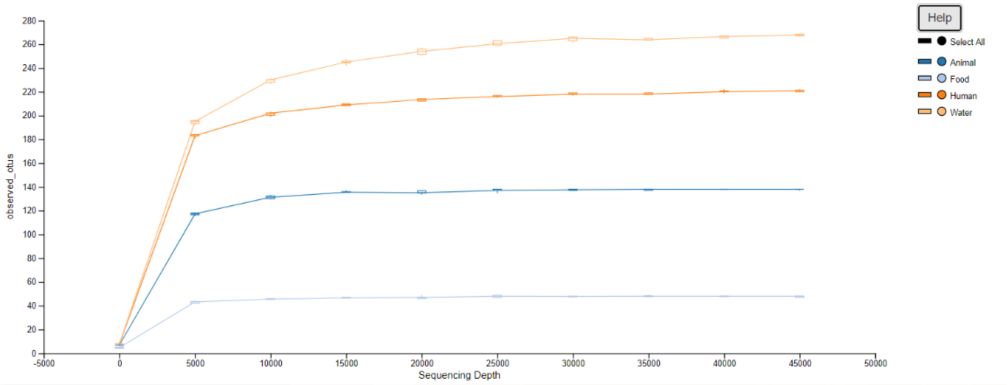

Figure S8: Sample-based rarefaction curves depicting the number of observed ASVs according to sequencing depth. Each line represents the curve of a different sample type. Light yellow line indicates the rarefaction curve of water samples, dark yellow line indicates the rarefaction curve of human stool samples, dark blue line indicates the rarefaction curve of animal stool samples, light blue line indicates the rarefaction curve of food samples.
